# Physioxia Expanded Bone Marrow Derived Mesenchymal Stem Cells Have Improved Cartilage Repair in an Early Osteoarthritic Focal Defect Model

**DOI:** 10.3390/biology9080230

**Published:** 2020-08-17

**Authors:** Girish Pattappa, Jonas Krueckel, Ruth Schewior, Dustin Franke, Alexander Mench, Matthias Koch, Johannes Weber, Siegmund Lang, Christian G. Pfeifer, Brian Johnstone, Denitsa Docheva, Volker Alt, Peter Angele, Johannes Zellner

**Affiliations:** 1 Laboratory of Experimental Trauma Surgery, Department of Trauma Surgery, University Hospital Regensburg, Franz Josef Strauss Allee 11, 93053 Regensburg, Germany; jonas.kreuckel@ukr.de (J.K.); ruth.schewior@ukr.de (R.S.); dustinfranke@web.de (D.F.); alexander.mench@ukr.de (A.M.); matthias.koch@ukr.de (M.K.); johannes1.weber@ukr.de (J.W.); siegmund.lang@ukr.de (S.L.); christian.pfeifer@ukr.de (C.G.P.); denitsa.docheva@ukr.de (D.D.); volker.alt@ukr.de (V.A.); angele@sporthopaedicum.de (P.A.); johannes.zellner@ukr.de (J.Z.); 2 Department of Orthopaedics and Rehabilitation, Oregon Health & Science University, 3181 SW Sam Jackson Park Rd, OP31, Portland, OR 97239, USA; johnstob@ohsu.edu; 3 Sporthopaedicum Regensburg, Hildegard von Bingen Strasse 1, 93053 Regensburg, Germany; 4 Department of Trauma Surgery, Caritas Hospital St. Josef, Landshuter Strasse 65, 93053 Regensburg, Germany

**Keywords:** mesenchymal stem cells, chondrogenesis, hypoxia, cartilage, early osteoarthritis

## Abstract

Focal early osteoarthritis (OA) or degenerative lesions account for 60% of treated cartilage defects each year. The current cell-based regenerative treatments have an increased failure rate for treating degenerative lesions compared to traumatic defects. Mesenchymal stem cells (MSCs) are an alternative cell source for treating early OA defects, due to their greater chondrogenic potential, compared to early OA chondrocytes. Low oxygen tension or physioxia has been shown to enhance MSC chondrogenic matrix content and could improve functional outcomes of regenerative therapies. The present investigation sought to develop a focal early OA animal model to evaluate cartilage regeneration and hypothesized that physioxic MSCs improve in vivo cartilage repair in both, post-trauma and focal early OA defects. Using a rabbit model, a focal defect was created, that developed signs of focal early OA after six weeks. MSCs cultured under physioxia had significantly enhanced in vitro MSC chondrogenic GAG content under hyperoxia with or without the presence of interleukin-1β (IL-1β). In both post-traumatic and focal early OA defect models, physioxic MSC treatment demonstrated a significant improvement in cartilage repair score, compared to hyperoxic MSCs and respective control defects. Future investigations will seek to understand whether these results are replicated in large animal models and the underlying mechanisms involved in in vivo cartilage regeneration.

## 1. Introduction

Articular cartilage lines the surfaces of synovial joints and facilitates loading and friction-free joint movement during locomotion [1]. Following traumatic injury, newly formed tissue is fibrocartilaginous and has different structural, biochemical and mechanical properties, compared with normal articular cartilage. The newly formed tissue remains susceptible to progressive degeneration that can lead to a significant reduction in a patient’s physical function. This degenerative disorder is known as osteoarthritis (OA) and does not solely affect articular cartilage, but all structures within the joint [2,3].

According to the Deutsche Gesellschaft fur Orthopaedie und Unfallchirurgie (DGOU) registry between October 2013 and June 2014, 60% of treated defects were degenerative. In a multi-centre study from 400 patients, approximately 35% had chondral lesions resulting from degenerative conditions [4,5]. Current strategies for the treatment of cartilage lesions have involved the use of surgical intervention (e.g., microfracture) or cell-based therapies [6]. Two cell-based products currently used in clinical practice are autologous chondrocyte implantation (ACI) or matrix-assisted chondrocyte transplantation (MACT). However, cell-based therapies for this condition have an increased failure rate compared with traumatic cartilage defects. A recent publication examining long-term follow-up (15 years) of OA patients treated with MACT, showed that there was a worsening of patient clinical scores (e.g., Tegner, EuroQol visual analog scale) with time and a great majority of patients (60%), either underwent re-operation or experienced clinical failure [7]. A reason for the poor clinical outcome is due to the inflammatory environment created, specifically the presence of the cytokine, interleukin-1β (IL-1β), correlated with poor MACT outcomes post-transplantation [5,8]. Due to the high proportion of degenerative cartilage lesions that require therapeutic intervention, regenerative options are required to overcome this challenging situation.

Recent studies have begun to focus on alternative cell sources for treating degenerative cartilage lesions. Mesenchymal stem cells (MSCs) are a potential cell source due to their multipotent differentiation (osteogenic, adipogenic and chondrogenic) and can be harvested from a variety of tissue sources (e.g. bone marrow, adipose (lipoaspirate from liposuction or Hoffa fat pad) or synovium tissue) with minimal donor site morbidity [9,10,11]. MSC chondrogenesis is induced via high density pellet or micromasses in the presence of pro-chondrogenic growth factors (e.g. transforming growth factor-beta (TGF-β)) to develop a cartilaginous matrix [12]. Studies have developed scaffolds/biomaterials that allow seeding or encapsulation of cells to create cartilaginous grafts for clinical translation [13,14]. A more robust cartilage matrix is formed from MSCs compared to articular chondrocytes, as the latter require extensive expansion to produce the requisite numbers of cells for clinical application. Furthermore, chondrocytes upon redifferentiation produce fibrocartilaginous tissue after extensive expansion, in contrast to MSC chondrogenesis [15]. However, the implantation of cartilaginous MSC grafts in nude mouse models has resulted in ectopic bone formation in the long-term [16,17].

Therefore, environmental stimuli that stabilize the cartilage phenotype are required, prior to implantation. Examples include mechanical loading (e.g., hydrostatic pressure, compression) or biochemical (e.g., oxygen tension, growth factors) factors [18,19]. Studies have extensively investigated the use of low oxygen tension or physioxia in both chondrocytes and MSC chondrogenesis (reviewed in Pattappa et al., 2019 [18]). Chondrocytes reside under a low oxygen environment (2–5% oxygen) in vivo, with a low oxygen gradient developed from the superficial to deeper regions of the tissue [20,21]. These low oxygen conditions are nominally termed as hypoxia. Recent publications have used the term, physioxia, in reference to the physiological oxygen tension that is far below atmospheric oxygen tension (20% oxygen). For the purposes of this publication, physioxia refers to low oxygen culture, whilst 20% oxygen is known as hyperoxia. Upon in vitro culture under physioxia, MSCs have been shown to significantly upregulate chondrogenic genes (SOX9, COL2A1 and ACAN) that lead to increased collagen II and GAG content within pellets or scaffolds. However, recent studies have demonstrated that the beneficial effects of physioxia on chondrogenesis is a donor dependent response [22,23]. Subsequent in vivo implantation of physioxia preconditioned chondrogenic MSCs into nude mouse and rabbit models resulted in increased cartilaginous matrix formation (GAG and collagen II) with reduced bone formation [24,25]. In contrast, there was no difference in cartilage matrix formation between hyperoxic and physioxic preconditioned chondrogenic MSCs in a sheep model [26].

Previous animal models for OA have primarily used either anterior cruciate ligament (ACL) resection, destabilized medial meniscus (DMM) or intra-articular enzymatic injection [27,28]. These models create diffusive OA that is difficult to treat and does not reflect the clinical scenario that utilize cell-based regenerative therapies. One aim of this study was to develop a model for focal early OA that induced a focal defect with no joint instability or changes to surrounding tissues in the joint (e.g., meniscus or ACL). Using this model, we evaluated the efficacy of physioxia expanded/physioxic MSCs in cartilage regeneration. It was hypothesized that physioxic MSCs would have improved cartilage regeneration compared with hyperoxic MSCs in both post-traumatic and focal early OA cartilage defects.

## 2. Materials and Methods

### 2.1. Animals and Study Design

The animal experiments used in the study were approved by the local ethics committee (Regierung von Unterfranken, 55.2 2532-2-300, approval date: 23 December 2016). The study was performed according to the approved guidelines and regulations. Thirty-five male New Zealand White rabbits (aged 5–6 months old) were used for the animal experiments. The groups and number of animals described in each study are described in Table 1. All animals were housed in single-animal cages in an air-conditioned environment under a 12 h/12 h day/night rhythm for the whole study period with free access to food and water.

### 2.2. In Vitro Rabbit Bone Marrow Culture

Bone marrow derived MSCs were extracted from the iliac crest of skeletally mature New Zealand white rabbits (*n* = 8; 5–6 months old) following anesthesia that was applied intramuscularly using a combination of 0.6 mL/kg ketamine (10%) and xylazine (2%). The bone marrow was harvested from the iliac crest via a small incision into the bone cortex with an 18G needle and collected into a heparin-containing syringe. Bone marrow was resuspended in fresh culture media, composed of low glucose Dulbecco’s Modified Eagle Medium (DMEM; Invitrogen, Karlsruhe, Germany) with 10% fetal bovine serum (FBS; PAN Biotech, Aidenbach, Germany), 1% penicillin/streptomycin (Invitrogen), 1% HEPES (Sigma-Aldrich, Steinheim, Germany)) and 5 ng/mL basic fibroblastic growth factor (bFGF; Peprotech, Hamburg, Germany). Bone marrow was centrifuged at 2000 rpm for five minutes and supernatant was carefully removed. The total number of nucleated cells was counted in the bone marrow aspirate and then seeded at a density of 2.5 × 10^5^ cells/cm^2^. In parallel, flasks were cultured, either in a standard cell culture incubator (20% oxygen, 5% CO_2_ and 70% N_2_) or a low oxygen incubator (2% oxygen, 5% CO_2_, 93% N_2_). This manuscript uses terminology stated in our previous manuscript, whereby 2% oxygen is known as physioxia, whilst 20% oxygen is described as hyperoxia [23]. The first media change was performed at five days post-seeding and then subsequent media changes were conducted twice a week.

Cells were trypsinized at 90% confluence and then cells counted using a hemacytometer. MSCs were cultured under their designated oxygen conditions for a further four passages with the data used to generate population growth curves. At each passage, photomicrographs were taken and images were analysed for cell area using ImageJ software (National Institutes of Health, Bethesda, MD, USA). In each image, ten individual cells from each image per condition and donor were evaluated.

### 2.3. In Vitro Chondrogenic Differentiation

At passage **2**, donor paired hyperoxic and physioxic MSCs (*n* = 8) were used to create pellet cultures, as previously described [23]. In brief, 4 × 10^5^ MSCs were formed in polypropylene V-bottom 96-well plates by centrifugation at 250× *g* for 5 min in 300 μL chondrogenic medium that consisted of serum-free high-glucose DMEM containing 10 ng/mL TGF-β1 (R&D systems), 100 nm dexamethasone, 50 µg/mL ascorbic acid-2-phosphate (all Sigma-Aldrich, Steinheim, Germany), 1 mM sodium pyruvate (Invitrogen) and 1% ITS (PAN Biotech GmbH, Aidenbach, Germany). A set of pellets were cultured in the presence of 0.1 ng/mL IL-1β (Peprotech), based on previous work [29]. Pellets were then cultured under their respective expansion oxygen conditions for 21 days with media changes performed every 2–3 days. In the case of physioxia pellets, media was pre-equilibrated in a physioxia incubator prior to replenishment.

### 2.4. Wet Weight and GAG Content Measurement

After 21 days in culture, the wet weight of pellets was measured using a balance. Macroscopic images of pellets were photographed with an optical microscope (PL2000, Optech, Germany). Triplicate pellets from each group were digested with 150 µg/mL papain in PBS, pH 6.0, containing 8 mM sodium EDTA, 6 mM L-cysteine (all Sigma-Aldrich). Digested pellet GAGs were quantified against a standard curve generated using bovine chondroitin sulphate A (Sigma-Aldrich) diluted in papain buffer as standard in serial dilution. DMMB dye (18 µg/mL in 0.5% ethanol, 0.2% formic acid, 30 mM sodium formate, pH 3) was added to standards and samples and absorbance measured at 575 nm (Tecan, Crailsheim, Germany).

### 2.5. Focal Early OA Model Development

**Figure 1:**
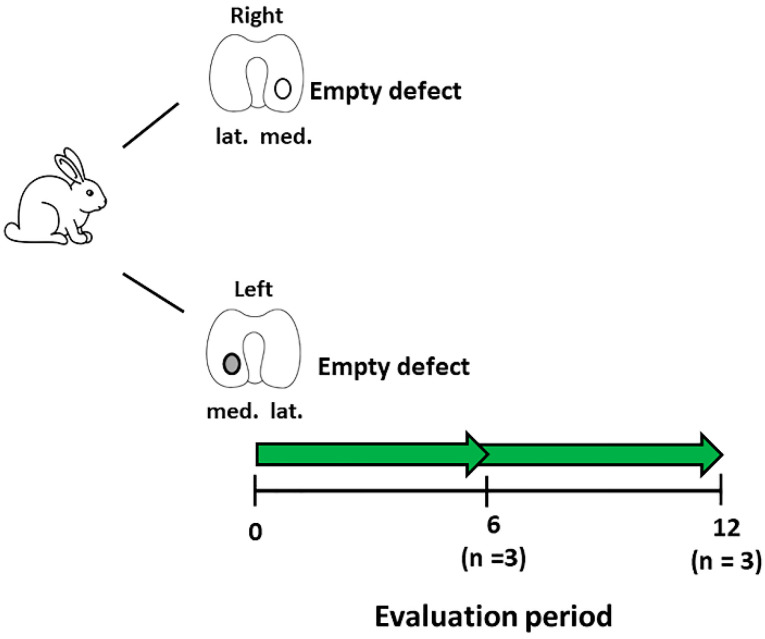
Schematic diagram describing the experimental setup for the development of the focal early OA model. Number of animals (*n*) in each group is stated in brackets.

The in vivo study was conducted on skeletally mature New Zealand white rabbits (5–6 months old) using procedures approved by our local ethics committee (Regierung von Unterfranken, 55.2 2532-2-300, approval date: 23 December 2016). The rabbits were anesthetized using a combination of 0.6 mL/kg ketamine (10%) and xylazine (2%), applied intramuscularly into the rabbit. The first animal study involved the development of a focal early OA model for the evaluation of cell-based therapies. Following anaesthesia, the medial femoral condyle was exposed through a medial parapatellar incision with the patella dislocated to the lateral side. A 2.3 mm diameter (2 mm deep) defect was created on the medial condyle using a handheld dental drill (Surgic Pro, NSK Europe, Eschborn, Germany). The patella was placed back into position and then skin was closed using sutures. A second defect was created on the contralateral medial femoral condyle using the same procedure (Figure 1).

The defects were left empty to observe the development of OA with time. Post-operatively, the rabbits were placed into their cages with no restrictions on movement and weight-bearing. To control pain, carprofen (5 mg/kg) was administered for five days post-surgery. Rabbits were sacrificed at 6 (*n* = 3) or 12 (*n* = 3) weeks post-defect creation. Histological sections were created from these defects, stained for Safranin-O/fast green (Section 2.7) and then were evaluated for OA using the OARSI score for rabbits (Section 2.8) by three blinded observers [30,31]. The time-point that was evaluated histologically to show signs of focal early OA was used for subsequent MSC treatment studies. For these studies, this model is defined as a focal early OA model, whilst defects treated immediately after creation are known as a post-trauma model.

### 2.6. IL-1β Measurement in Cartilage

At the time of sacrifice for evaluating the focal early OA model, cartilage pieces surrounding the defect were removed using a scalpel and then frozen for analysis. Cartilage pieces were washed in ice cold PBS and homogenized in 200 μL 8 M urea/2% SDS solution using a PreCellys homogenizer (Bertin Instruments, Montigny le Bretonneux, France). The lysate was centrifuged for 5 min at 1000× *g* (4 °C) and the supernatant was transferred to a fresh tube. The protein concentration of the supernatant was determined using the BCA Protein Assay kit (Biorad, DC Protein Assay, Hercules, CA, USA) according to the manufacturer’s instructions. ELISA detection for rabbit IL-1β was performed according to manufacturer’s protocol (rabbit IL-1β; R and D systems, UK).

### 2.7. MSC Treatment for Focal Early OA and Post-Trauma Models

**Figure 2:**
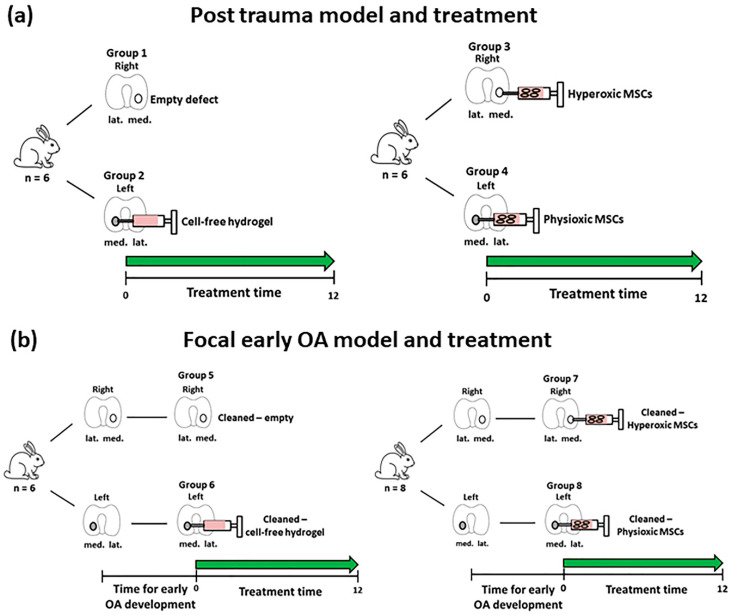
Schematic diagram describing the defect models of (**a**) Post-trauma and (**b**) focal early OA with the respective treatment groups for these models. Number of animals (*n*) in each group is stated in brackets.

Rabbits were grouped into either post-trauma (groups 1–4; Figure 2a) or focal early OA (groups 5–8; Figure 2b) models. The post-trauma defect model is described as a focal defect that is created and immediately treated (Figure 2a), while the focal early OA defect is a focal defect that is generated, and then left to develop signs of early OA around the edges of the defect, over a designated time period (results from Section 2.5), and then treated (Figure 2b).

For the post-trauma model (Figure 2a), defects were created on both medial condyles of right and left knee of each rabbit and then either left empty (group 1; *n* = 6 rabbits) or injected with a commercially available hyaluronic acid hydrogel (TETEC, Tübingen, Germany) [32] on the contralateral medial femoral condyle without cells (group 2; *n* = 6). In the case of the focal early OA model (Figure 2b), two surgeries were performed, the first involved defect creation as described and then following development of focal early OA, a second surgery was undertaken to clean the defect using a 22G needle to remove repair tissue. This was then either left empty (Group 5; *n* = 6), or injected with the described cell-free hydrogel (Group 6; *n* = 6) on the controlateral medial femoral condyle.

In the case of MSC treated defects for both models, autologous rabbit MSCs (passage 2; *n* = 26, including donors from the in vitro study (Section 2.2)) isolated from bone marrow were pre-expanded in parallel under either hyperoxia or physioxia (see Section 2.2). MSCs were counted using a haemacytometer and seeded into the hydrogel at a density of 50 × 10^6^ cells/mL according to a previously described protocol [32]. The cell seeding density was based on previous in vivo experiments using MSCs for cartilage repair in animal models [33,34]. MSC-hydrogels were injected into the defect (approx. 20 μL) with one medial femoral condyle treated with physioxic MSCs, whilst the contralateral medial femoral condyle had hyperxoic MSCs applied (Figure 2). Prior to joint closure, all hydrogel groups were allowed to gel for five minutes and joint motion was used to observe whether hydrogel remained in the defect. For post-trauma defects, hyperoxic MSCs (Group 3; *n* = 6) and physioxic MSCs (Group 4; *n* = 6) were immediately injected following defect creation (Figure 2a). In the focal early OA model, hyperoxic (Group 7; *n* = 8) and physioxic (Group 8; *n* = 8) MSCs were applied in a second surgery, following the designated period time for focal early OA development (Figure 2b). All rabbits were put back into their individual cages with no restrictions on movement. Prior to these experiments and to evaluate whether hydrogel remained within the defect, a pilot study was conducted, whereby defects were created on both knees as described and then immediately implanted with cell-free hydrogel. The rabbits were euthanized at 12 weeks post-MSC implantation for the respective model (for the pilot study, only 1 week post hydrogel application) by an intravenous overdose application of narcoren (0.5 g/kg).

### 2.8. Decalcification and Histology/Immunohistochemistry

Femoral condyles were extracted from the rabbits and were fixed in 4% paraformaldehyde (PFA) for 48 h at 4 °C. Condyles were rinsed in 0.1M phosphate buffer and decalcified in 10% Ethylenediaminetetraacetic acid (EDTA; pH 8) in PBS. EDTA solution was changed three times a week and condyles placed on a manual shaker. Following decalcification, condyles were incubated with increasing sucrose concentrations (10–30%) and then embedded in Tissue-Tek (Sakura, Zoeterwoude, The Netherlands). Embedded condyles were cryosectioned at 8 µm using a cryostat (Leica CM 1950, Leica Biosystems, Germany). MSC chondrogenic pellets were embedded using the same protocol without the requirement for decalcification.

Sections of the defect region were stained with safranin-O (0.1% *w/v*)/Fast green (0.05% *w/v*) (Sigma Aldrich, Steinheim, Germany) to stain sulphated glycosaminoglycans and collagens in the embedded condyles. For chondrogenic pellets, sulphated glycosaminoglycan content was detected by histochemical staining with DMMB (0.05% 1, 9-dimethylmethylene blue, 0.5% ethanol, 0.2% formic acid, 30 mM sodium formate, pH 3). Sections used for immunohistochemistry were rehydrated and antigen retrieval was performed at room temperature. To observe whether hyaline cartilage is formed (high collagen II staining) or fibrocartilage was formed (staining for collagen I and II), immunohistochemical staining for collagen I and II was performed. For collagen I and collagen II (both Calbiochem, Darmstadt, Germany), sections were treated with 3 mg/mL pepsin (Sigma-Aldrich) in 1xcitric/phosphate McIlvaine buffer for 15 min. Sections were blocked with a blocking buffer (10% goat serum in 1xPBS) and then incubated overnight with primary antibody to probe for rabbit collagen I (mouse monoclonal, 1:100) and collagen II (mouse monoclonal, 1:100). Following primary antibody incubation, biotinylated secondary antibody (goat anti-mouse IgG, 1:100) was applied and positive staining was visualized using nickel- and cobalt-enhanced 3, 3′-diaminobenzidine (DAB).

### 2.9. Cartilage Scoring

Three blinded observers with prior knowledge of cartilage repair and histological assessment scored five safranin-O/fast green stained sections per condyle. Defect only sections were evaluated for cartilage degeneration using the OARSI histological score for rabbits (Table S1) [30,31]. For cartilage regeneration, control (defect only, cell-free hydrogel) and MSC treated defects were scored using the Sellers scoring system (Table S2) [35,36]. This is an inverse scoring system, whereby the lower the score, the better the cartilage repair.

### 2.10. Statistical Analysis

All statistical analysis was performed using Graphpad Prism v7 (GraphPad, La Jolla, CA, USA). A Shapiro-Wilk normality test was used to test for normality of the data with *p* < 0.05, indicating use of non-parametric statistical test. Pellet wet weight and GAG content was analyzed using two-way ANOVA with Tukey post-hoc test. Individual Sellers score parameters were tested for significance using a Kruskal-Wallis test with Dunn’s post hoc test. Comparison between OARSI and total Sellers score used a One way ANOVA with Tukey post-hoc test. A pairwise t-test was used to compare Sellers score between hyperoxia and physioxia MSC treated defects in both animal models. Correlation analysis between in vitro per pellet GAG and in vivo Sellers score outcome was conducted using Pearson correlation co-efficient test. Significance was set at * *p* < 0.05.

## 3. Results

### 3.1. Development of A Focal Early OA Model

**Figure 3:**
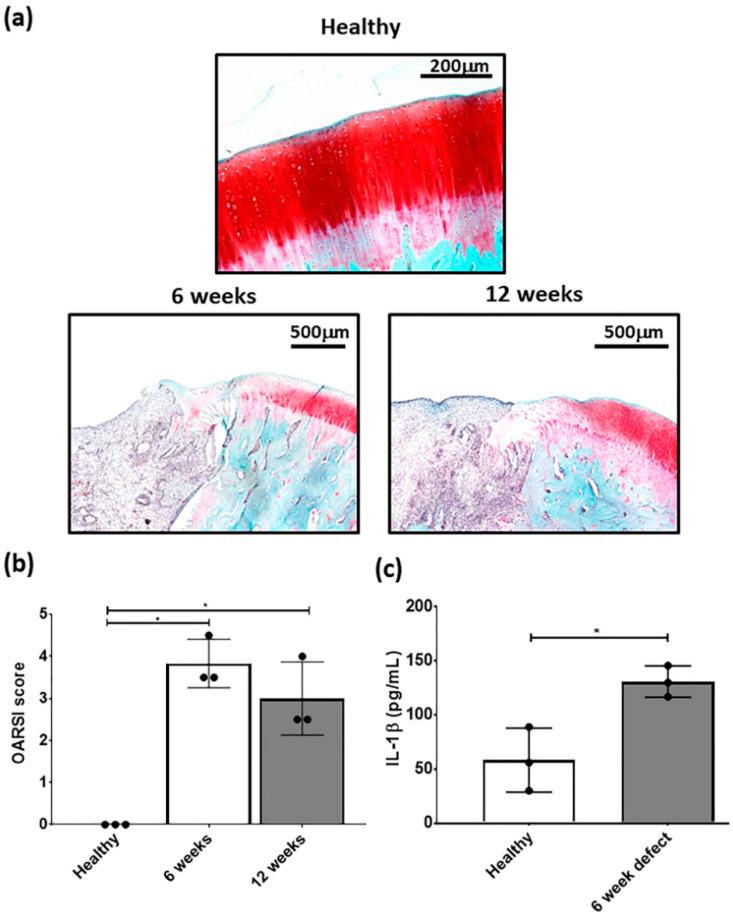
Representative (**a**) safranin-O/fast green stained healthy, 6 and 12 week defects used for the examination of focal early OA in the defect. (**b**) OARSI score for healthy, 6 and 12 week defects post-surgery and (**c**) measurement of IL-1β in cartilage from healthy and defect cartilage. Data represent mean ± S.D.; *n* = 3, with dots representing the mean from three blinded scorers (five sections per defect). * *p* < 0.05.

Focal osteochondal defects with a diameter of 2.3 mm and a depth of 2 mm were created on the posterior portion of the medial femoral condyle. At both, 6 and 12 weeks after defect creation, gross evaluation showed areas of degeneration with cartilage abrasion and softening in the region around the defect in the medial compartment. Histological examination showed that the structure of the cartilage layer adjacent to the defect had evidence of cell death, clustering and proliferation with fissures formed in the tissue. In the area of the defect, there were no signs of cartilaginous tissue with no safranin-o/fast green staining (Figure 3a).

OARSI scoring of the defect had a mean score between 3 and 4 at 6 and 12 weeks post-defect creation and was significantly different from healthy cartilage (Figure 3b). Furthermore, there was a significant elevation in IL-1β concentration in the cartilage adjacent to the defect at 6 weeks post-defect (Figure 3c). Based on this data, subsequent focal early OA experiments with the respective treatments, were carried out at 6 weeks post defect creation. Although, both time points showed signs of focal early OA, the 6 week time point had the earliest signs of the condition and was used for subsequent MSC studies.

### 3.2. Rabbit MSCs Undergo Greater Population Doublings and Have Enhanced Chondrogenesis Under Physioxia

**Figure 4:**
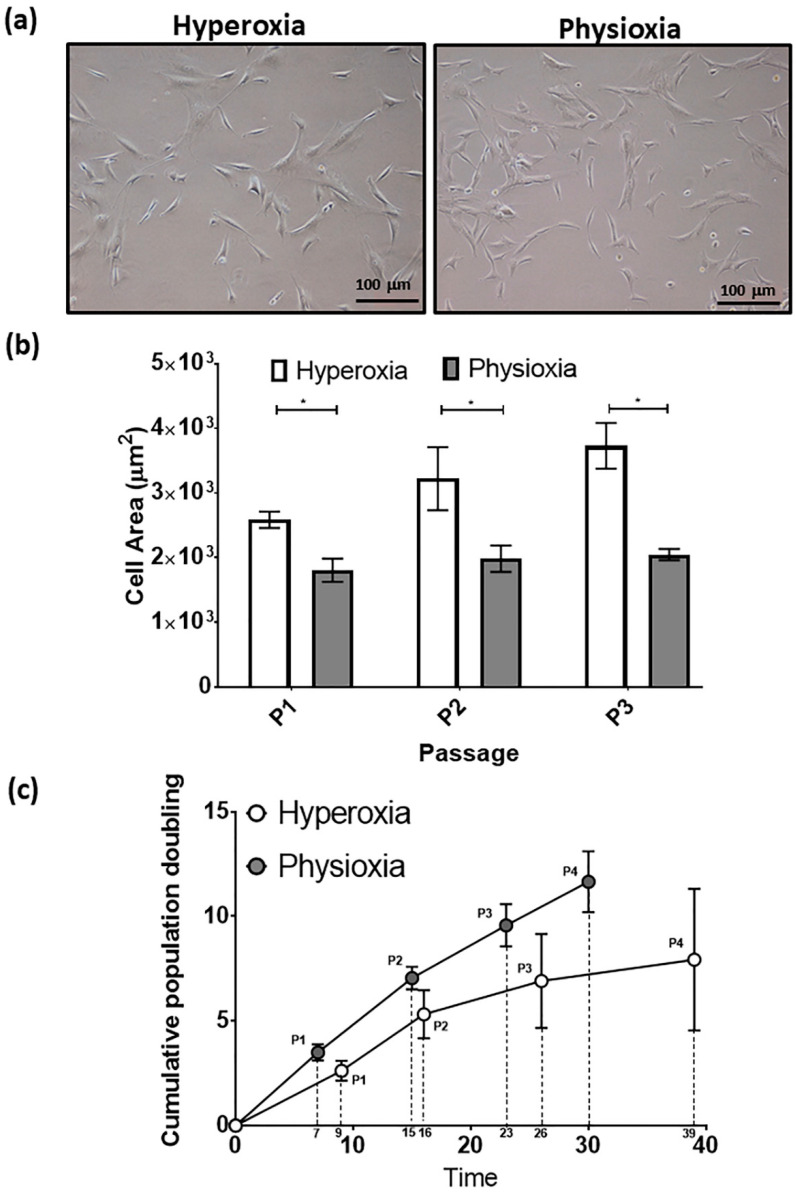
(**a**) Photomicrographs of rabbit MSCs cultured under hyperoxia and physioxia. (**b**) Cell area measurements for individual MSCs from photomicrographs (10 individual cells measured per image) and (**c**) cumulative cell growth of MSCs under physioxia and hyperoxia. Data represent mean ± S.D.; *n* = 8; * *p* < 0.05.

The typical MSC fibroblastic morphology was observed under both oxygen tensions, with MSCs cultured under physioxia appearing to be smaller (Figure 4a) [37]. Measurements of individual MSCs demonstrated that physioxic MSCs were significantly smaller than hyperoxic MSCs (*p* < 0.05; Figure 4b). Furthermore, hyperoxic MSCs increased their cell area with passage, whilst physioxic MSCs remained the same. An analysis of their cellular proliferation over four passages in culture, showed that physioxic culture resulted in greater MSCs numbers and significantly shorter population doubling times between passages relative to hyperoxia (* *p* < 0.05; Figure 4c). In the case of hyperoxic MSCs, there was a cessation of cell growth after approximately 30 days in culture in the majority of donors, whereas physioxic MSCs continued to expand at the same rate for all donors (Figure 4c).

**Figure 5:**
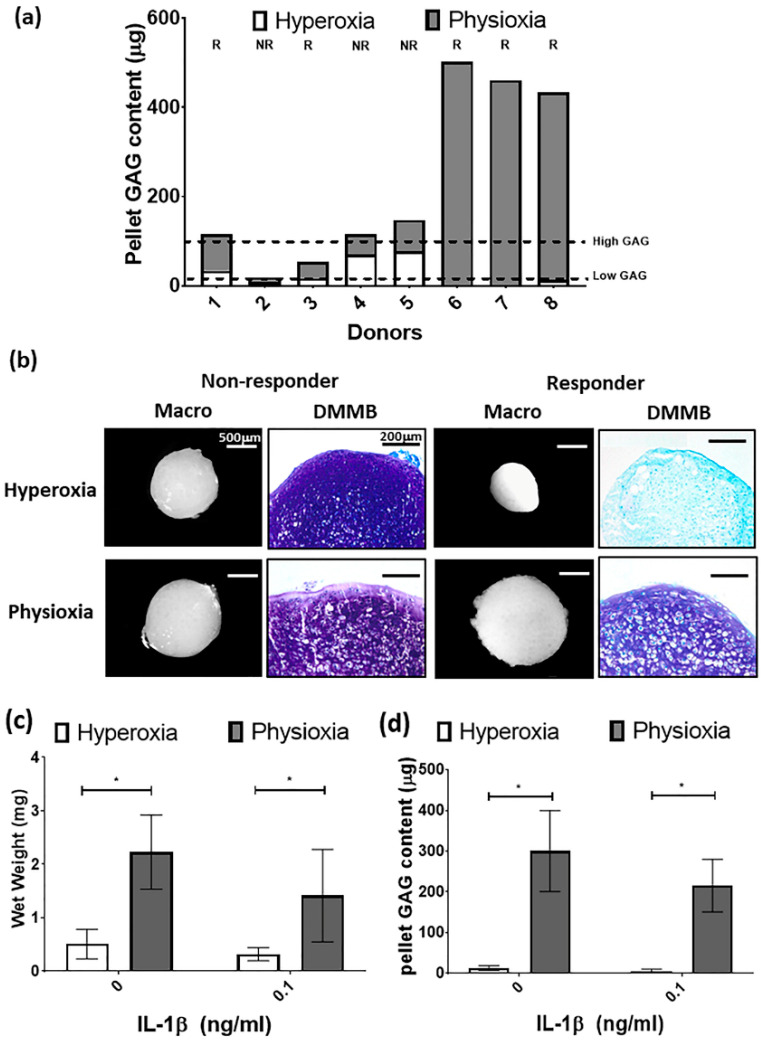
(**a**) Pellet GAG content under physioxia and hyperoxia for chondrogenic pellets. Dotted lines represent threshold for high (physioxia non-responsive) and low (physioxia responsive) GAG donors. R represents physioxia responders and NR are physioxia non-responders. (**b**) Representative macroscopic and DMMB stained chondrogenic pellets of physioxia non-responder and responder pellets. Pellet (**c**) wet weight; and (**d**) GAG content for physioxia responders with or without the presence of IL-1β. Data represent mean ± S.D.; *n* = 5. * *p* < 0.05.

For the purpose of the subsequent in vivo studies, MSC chondrogenesis was performed for donors under their expansion conditions to evaluate their response to physioxia. There were donors either responsive (R) or non-responsive (NR) to physioxia based upon their pellet GAG content (Figure 5a). A physioxia responsive donor is one where there was a 2-fold increase in pellet GAG under physioxa relative to hyperoxia, whereas a non-responder was one that a had a less than 2-fold increase [23]. Additionally, these responsive donors had a low intrinsic baseline chondrogenesis under hyperoxia [22]. Histological evaluation of non-responsive donors showed pellets of similar size and DMMB staining intensity. In contrast, physioxia responsive donors had larger pellets and greater DMMB staining intensity under physioxia compared to hyperoxic cultures (Figure 5b). Analysis of only physioxia responsive donors demonstrated a significant increase in pellet wet weight (* *p* < 0.05; Figure 5c) and GAG content (* *p* < 0.05; Figure 5d) under these conditions, when compared to hyperoxic culture. In the presence of IL-1β, a decrease in pellet wet weight and GAG content was observed under hyperoxic conditions. However, the corresponding physioxic condition, demonstrated a significant increase in pellet wet weight and GAG content compared to equivalent hyperoxic cultures (* *p* < 0.05; Figure 5b,c).

### 3.3. Physioxic Preconditioned MSCs Support An Improvement in Cartilage Repair in A Post-Trauma Cartilage Defect

**Figure 6:**
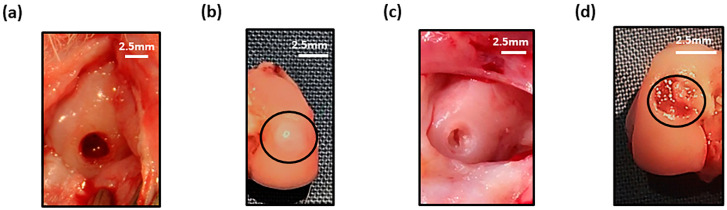
Macroscopic image of (**a**) empty defect immediately after creation and at (**b**) 6 weeks (defect cricled) post-surgery. (**c**) Cleaned defect prior to hydrogel implantation and (**d**) presence of hydrogel (black circle) in defect after one week in the joint.

Defects on the medial femoral condyle (Figure 6a) that were left empty after six weeks showed tissue repair with filling of the defect (Figure 6b). For the focal early OA model, the defect was cleaned using a 22G needle to remove the repair tissue (Figure 6c), prior to application of cell-free or MSC-hydrogel into the defect. A pilot study demonstrated that injected hydrogel stayed within the defect after one week post-implantation (Figure 6d).

**Figure 7:**
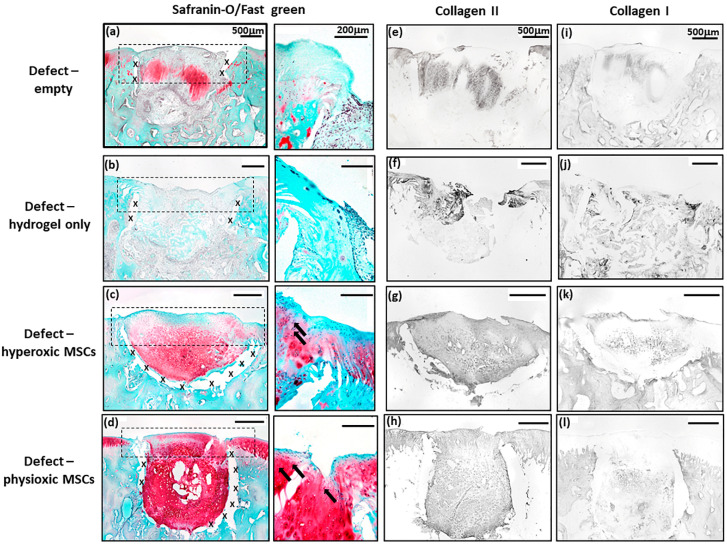
Representative images of (**a**–**d**) Safranin-O/Fast green, (**e**–**h**) collagen II and (**i**–**l**) collagen I staining for (**a**,**e**,**i**) empty defect, (**b**,**f**,**j**) hydrogel only, (**c**,**g**,**k**) hyperoxia and (**d**,**h**,**l**) physioxia treated post-trauma defects at 12 weeks post treatment. Box dotted line represents cartilage region evaluated for Sellers score and cross (x) signifies artefacts in the section. Arrows depict chondrocytes/chondrocytic columns in the cartilage layer.

MSC treated defects had cartilaginous tissue formation with safranin-O and collagen II staining within the treated region. Application of physioxic MSCs induced greater safranin-O and collagen II staining (Figure 7d,h) with hyperoxic (Figure 7c,g). Furthermore, there was evidence of more chondrocytes and early column formation in the cartilage layer upon application of physioxic MSCs relative to hyperoxic MSCs (Figure 7c,d). In contrast, empty and cell-free hydrogel defects had fibrocartilaginous tissue with minimal safranin-o staining (Figure 7a,b) and only collagen II (Figure 7e,f) and I (Figure 7i,j) present.

**Figure 8:**
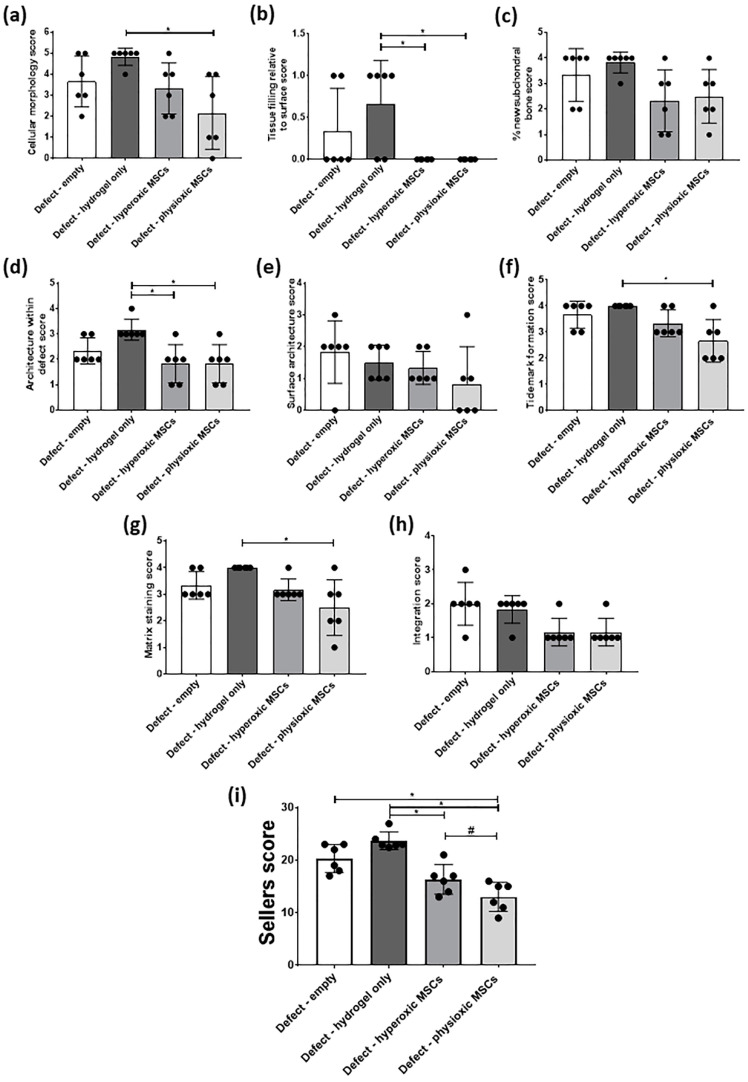
Individual scoring parameters of the Sellers score for (**a**) cellular morphology, (**b**) tissue filling, (**c**) % new subchondral bone, (**d**) architecture within the defect, (**e**) surface architecture, (**f**) new tidemark formation (**g**) new cartilage matrix staining and (**h**) cartilage integration with surrounding cartilage between groups in the post-trauma model. * *p* < 0.05 from Kruskal-Wallis test with Dunn’s multiple comparison test. (**i**) The overall Sellers score for cartilage repair in a post trauma model). * *p* < 0.05 from one way ANOVA with Tukey post-hoc test, ^#^ *p* < 0.05 from pairwise t-test. Data represent the mean ± S.D. of *n* = 6 rabbits with dots representing the mean from three blinded scorers (five sections per defect).

In general, scoring of cartilage regeneration showed improved scores for physioxic MSC treated defects (Figure 8). Assessment of individual parameters of the Sellers score showed a significant improvement in cellular morphology, new tissue filling, matrix staining, tissue architecture within the defect and new tidemark formation with physioxic MSCs compared to hydrogel only treated defects (* *p* < 0.05; Figure 8a,b,d,f,g). No significant differences in score were found for tissue integration, percentage new subchondral bone formation or surface architecture, although scores were lower for MSC treated defects (Figure 8c,e,h).

The total Sellers score was found to have a lower score for MSC treatments compared with empty and cell-free hydrogel treated defects. Both hyperoxic and physioxic MSCs showed a significant improvement in the score compared to the controls (* *p* < 0.05; Figure 8i). Furthermore, physioxic MSCs resulted in a significant improvement in tissue regeneration, compared with hyperoxic MSCs (^#^ *p* < 0.05; Figure 8i).

### 3.4. Physioxic Preconditioned MSCs Demonstrate A Significant Enhancement in Cartilage Repair in A Focal Early Osteoarthritic Defect

**Figure 9:**
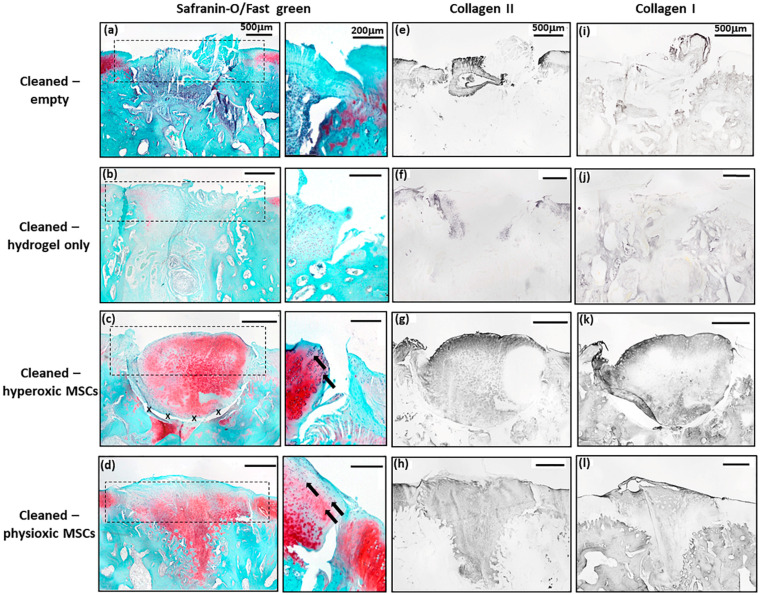
Representative images of (**a**–**d**) Safranin-O/Fast green, (**e**–**h**) collagen II and (**i**–**l**) collagen I staining for (**a**,**e**,**i**) empty defect, (**b**,**f**,**j**) hydrogel only, (**c**,**g**,**k**) hyperoxia and (**d**.**h**,**l**) physioxia treated focal early OA defects at 12 weeks post treatment. Box dotted line represents cartilage region evaluated for Sellers score and cross (x) signifies artefacts in the section. Arrows depict chondrocyte/ chondrocytic columns in the cartilage layer.

**Figure 10:**
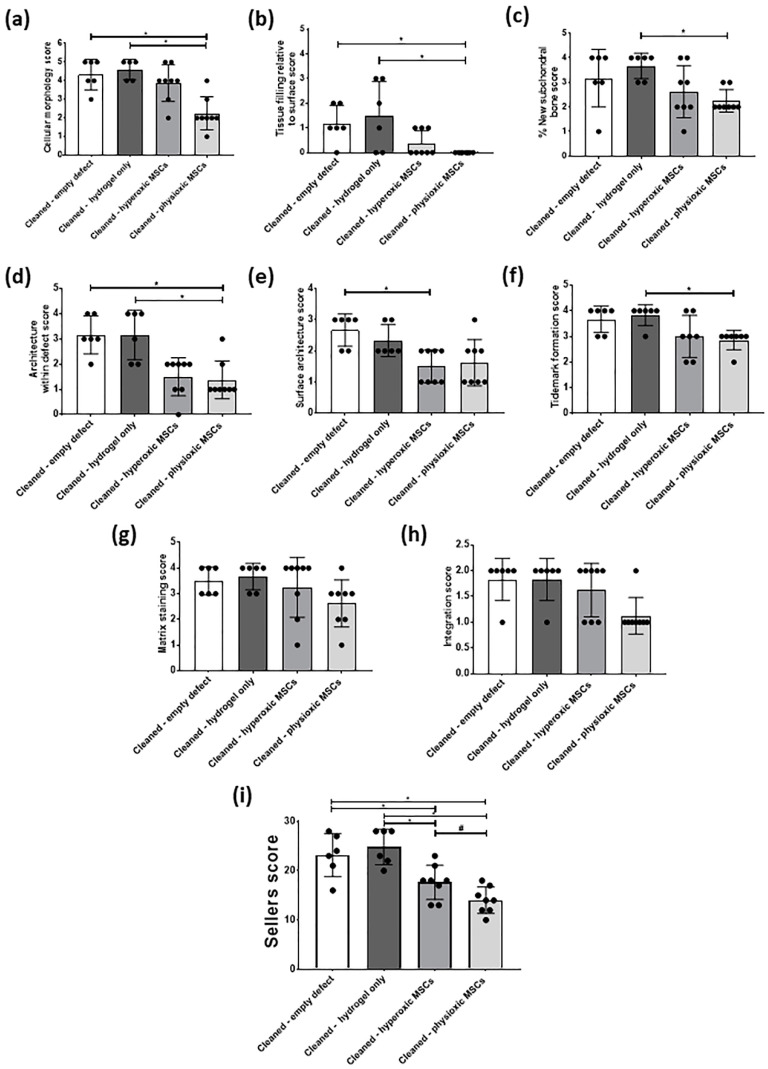
Individual scoring parameters of the Sellers score for (**a**) cellular morphology, (**b**) tissue filling, (**c**) % new subchondral bone, (**d**) architecture within the defect, (**e**) surface architecture, (**f**) new tidemark formation (**g**) new cartilage matrix staining, and (**h**) cartilage integration with surrounding cartilage between groups in a focal early OA model. * *p* < 0.05 from Kruskal-Wallis test with Dunn’s multiple comparison test. (**i**) The overall Sellers score for cartilage repair in a focal early OA model. * *p* < 0.05 from one way ANOVA with Tukey post-hoc test, ^#^ *p* < 0.05 from pairwise t-test. The data represent the mean ± S.D. of *n* = 6–8 rabbits with dots representing the mean from three blinded scorers (five sections per defect).

Similar to the post-trauma model, MSC application had more safranin-O and collagen II staining compared to empty and hydrogel only treated defects in a focal early OA model (Figure 9). Examination of physioxic MSCs treated defects showed chondrocytes and columns beginning to form in the cartilage layer, whilst hyperoxic MSC therapies had fewer chondrocytes or columns forming in this layer. No safranin-O (Figure 9a,b) and minimal collagen staining (Figure 9e,i,f,j) was observed in control defects. This observation corresponded to lower scores for MSC treatments independent of cultured oxygen tension compared to empty and cell-free hydrogel only defects (Figure 10). Specifically, cellular morphology, new tissue filling, percentage new subchondral bone formation, surface architecture, tissue architecture in the defect and tidemark formation were found to have a significant improvement with physioxic MSC application compared to control defects (* *p* < 0.05; Figure 10a–d,f). For hyperoxic MSCs, significant differences were only found for surface architecture compared with cleaned and empty defects (* *p* < 0.05; Figure 10e). No significant difference between groups was observed for new cartilage integration and matrix staining (Figure 10g,h).

There was found to be a significant reduction in Sellers score with MSC presence, independent of expanded oxygen tension compared to controls (cell-free hydrogel and empty) (* *p* < 0.05; Figure 10i). A pairwise comparison of MSC therapies showed a significant improvement using physioxic MSCs (^#^ *p* < 0.05; Figure 10i).

### 3.5. Paired Analysis Shows An Improvement in Cartilage Regeneration with Physioxic MSCs and A Correlation Between Sellers Score and Per Pellet GAG Content

**Figure 11:**
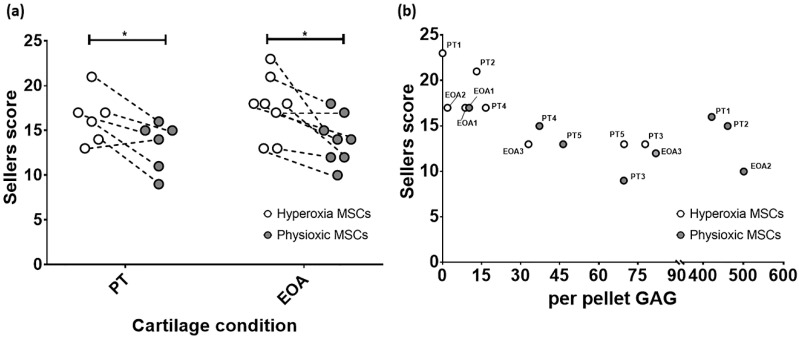
(**a**) Dot plot describing paired comparison between physioxia and hyperoxic MSCs treatment in post-trauma (PT) and focal early OA (EOA) models (* *p* < 0.05). (**b**) A dot plot depicting the relationship between per pellet GAG and subsequent Sellers score outcome for the treated defect. PT signifies post trauma model and EOA represents focal early OA model with respective animal number given.

Comparison of only MSC groups in either model, showed that in both post trauma (5/6) and focal early OA (7/8) models, a better Sellers score was achieved with physioxic MSCs compared with hyperoxic MSCs (* *p* < 0.05; Figure 11a). Plotting the in vivo Sellers score outcome against in vitro per pellet GAG content for those rabbit donors evaluated for both parameters, showed a tendency towards a lower Sellers score with increased per pellet GAG. However, this trend was not statistically significant (*p* > 0.05; Figure 11b).

## 4. Discussion

The present investigation sought to understand the effect of physioxic MSCs on cartilage repair in two different clinical scenarios, post trauma and focal early OA cartilage lesions. For the latter scenario, a focal defect was created using a dental drill that induced osteoarthritic-like changes at the defect margins after six weeks and met the criteria described for focal early OA (Figure 3) [38]. Treating defects with a hyaluronic acid hydrogel seeded with physioxic MSCs resulted in a significant improvement in cartilage repair scores compared with hyperoxic MSCs in both clinical scenarios (Figure 7, Figure 8, Figure 9 and Figure 10).

In vivo studies evaluating cell-based therapeutic strategies for cartilage repair have primarily focused on post-traumatic defects and late stage OA. In the latter, current animal models induce a diffusive OA throughout the joint through either intra-articular enzyme injection (e.g., collagenase or papain) or resection of joint structures (e.g., meniscus or ACL) [27,28]. However all these methods for OA induction are non-reversible, whereas the clinical target for cell-based regenerative approaches to OA are focal lesions rather than diffusive OA. Animal models that create a focal early OA lesion with no joint instability or changes in joint structures are needed to evaluate regenerative approaches. Existing focal OA models only induce a mild degeneration in the joint and they do meet the minimal criteria for early OA, e.g., impaction or groove models [39].

In the present investigation, a dental drill device was used to create a focal defect in the posterior portion of the medial femoral condyle due to the high biomechanical load applied during joint movement that would lead to a more degenerative lesion with time [40,41]. The strength of the model is that it induces a reproducible and consistent focal defect that develops into a focal early OA local to the surrounding area rather than throughout the defect. Furthermore, this model can be used to evaluate the efficacy of treating focal early OA lesions over time using methods that either stop or attenuate OA progression. At both time points evaluated, cell clustering and cellular proliferation was observed, whilst fissures formed in the cartilage layer (Figure 3a). OARSI scoring for the defect showed a degeneration that met the criteria for focal early OA (Figure 3b) [38]. The six week time point was defined as focal early OA, with joints showing signs of early OA development and elevated levels of IL-1β (Figure 3c). The latter is an indication of focal early OA, as its production leads to adverse clinical outcomes for ACI and MACT treatments in patients with focal and degenerative cartilage lesions [5,8]. A limitation of the use of rabbits for the focal early OA model is that they have different gait/biomechanics and cartilage biochemistry compared with humans. Furthermore, rabbits have a good capacity for spontaneous healing or repair of damaged cartilage due to their higher chondrocyte density compared to humans [27,42,43]. This was demonstrated in the present investigation by the fibrocartilaginous tissue formed in the empty defects in both models (Figure 7a,e,i and Figure 9a,e,i). Sheep or pigs have a thicker articular cartilage and similar biochemistry to humans, enabling the evaluation of focal early OA restricted to the chondral layer with a repair capacity similar to humans [27,42]. In this respect, Pfeifer et al. (2017) showed that partial chondral defects are achievable in juvenile and adult pigs [44]. Future studies would seek to create this focal early OA model into larger animals to better mimic the clinical scenario.

Physioxic MSCs had significantly greater cell numbers and enhanced proliferation compared with hyperoxic MSCs (Figure 4). This is in keeping with outcomes of previous studies examining human MSC expansion from different cell sources under physioxia [23]. There are two important features that support the use of physioxia for MSC expansion, the prevention of cellular senescence and preservation of MSC multipotency [45,46,47]. In the case of cellular senescence, this can be indicated by cellular morphology, as our present results show that hyperoxic MSCs increased their cell area with passage, whilst physioxic MSCs kept a constant cell area with passage (Figure 4b). This also led to reduced cell numbers with passage and in certain donors examined; there was a cessation of growth under hyperoxia, whilst physioxia maintained a steady growth in the donors examined (Figure 4c).

Previous studies investigating MSC differentiation under physioxia have shown that there is a donor dependent positive response in MSC chondrogenesis upon physioxic culture [22,23], whilst for osteogenic differentiation there is an inhibition in calcified matrix formation compared to hyperoxic culture [48,49]. In the present study, subsequent MSC chondrogenesis demonstrated a donor dependent response to physioxia with respect to pellet GAG content that has been previously described for human MSCs donors (Figure 5a) [22,23]. Anderson et al. (2016) and Pattappa et al. (2019) separated donors according to their response to physioxia based on their inherent GAG content. These thresholds for rabbit MSCs are shown in Figure 5a, whereby low GAG donors are physioxia responders and high GAG doors are physioxia non-responders. Analysis of only physioxia responsive rabbit MSC donors demonstrated a significant increase in pellet wet weight and pellet GAG content with or without the presence of IL-1β, correlating with results from previous human studies [23,50]. IL-1β was used to mimic an early OA situation in vitro. The mechanism controlling the response under inflammatory conditions has been hypothesized to be associated with TGF-β receptor I/II activity. IL-1β inhibits their activity under hyperoxia and prevents downstream TGF-β/SMAD pathways with a downregulation in cartilage matrix genes. Physioxia alleviates IL-1β inhibition, as TGF-β receptor I/II expression are upregulated under this condition, restoring downstream signaling pathways and subsequently upregulates expression of cartilage matrix genes [23,51,52,53]. Further mechanistic studies are required to understand how physioxia helps overcome an inflammatory environment in MSC chondrogenesis.

Implantation of physioxic MSCs into either post-trauma or focal early OA defects showed a significant improvement in cartilage repair score compared with hyperoxic MSCs (Figure 7, Figure 8, Figure 9 and Figure 10). In both scenarios, hydrogel only defects had the worst cartilage repair score, whilst there was some form of repair in empty defects (Figure 7a,b and Figure 9a,b). However, there was no safranin-O staining and only collagen I and II present. In comparison, MSC treated defects had greater safranin-O and collagen II staining. Specifically for physioxic MSC treated defects, there was evidence of chondrocytes and chondrocyte columns beginning to form in the cartilage layer (Figure 7c,d and Figure 9c,d). These morphological observations were also reflected in individual scores and the total Sellers score, as lower scores were recorded for MSC treated defects, especially in focal early OA treatments (Figure 8 and Figure 10). Paired comparison of only MSC treated defects revealed a significant improvement in both post-trauma and focal early OA defects with physioxic MSCs compared with hyperoxic MSCs (Figure 11a). In the donors examined for both in vitro per pellet GAG content and in vivo Sellers score, the majority of donors had a higher in vitro per pellet GAG content under physioxia and this also led to a lower Sellers scores following implantation in vivo. However, there was found to be no correlation between the parameters (Figure 11b). Other in vitro biomarkers to predict in vivo cartilage repair are to be examined in future investigations.

Previous studies investigating the effect of physioxia on in vivo cartilage repair have used physioxia expanded and chondrogenically differentiated MSCs prior to implantation in post trauma animal models. Portron et al. (2013) using this preconditioning model on adipose derived MSCs showed the best/highest O’Driscoll cartilage repair score was obtained by these cells compared with corresponding hyperoxic preconditioned chondrogenic MSCs, although there was no statistically significant difference between therapies. A recent study using the same preconditioned parameters with bone marrow derived MSCs in a sheep model, showed no difference in O’Driscoll score upon application of physioxia or hyperoxia preconditioned chondrogenic MSC in a post trauma cartilage defect [26]. The latter results could be due to the sustained physioxic environment generated from the thicker articular cartilage that is present within sheep. The reason for the differences in the present study could be related to only applying physioxic MSCs without chondrogenic induction into the animal and the thin cartilage layer of rabbit does not create a sustained physioxic environment. Thus, future studies in large animal models would investigate whether physioxic MSCs without predifferentiation would display similar differences in cartilage regeneration to the present study.

The histological evidence and cartilage repair scores indicate that regeneration is occurring with MSC treated defects; however, this is only neocartilage formation. Whether the MSC generated cartilage remains stable or leads to hypertrophy remains unknown. Previous studies in small animal models have indicated that physioxia expanded and chondrogenically differentiated MSCs reduced cartilage hypertrophy and bone formation upon in vivo implantation [24,25]. In our study, the implantation of MSCs was associated with greater subchondral bone formation within the defect, below the newly generated cartilage (Figure 8c and Figure 10c). A limitation of the study is that evaluation at 12 weeks (3 months) cannot provide an indicator for the long-term efficacy of physioxic MSC treated defects. Later time points at 6 and 12 months are required to fully understand whether the newly-formed cartilage tissue remains stable or eventually becomes bone with time.

The significant improvement of cartilage repair with MSC presence compared to cell-free hydrogel treatment shows the effectiveness of cell-based therapies for treating focal early OA or trauma lesions. Although implantation with physioxic MSCs shows promise in this and previous studies, there remains an open question concerning whether this is the appropriate cell type for cartilage regeneration. Recent investigations have described the presence of articular cartilage progenitor (ACPs) cells within the superficial zone of both healthy and OA articular cartilage [54,55]. In comparison to physioxic MSC chondrogenesis that still produces collagen X, inspite of a downregulation in hypertrophic markers (e.g., collagen X, MMP13), isolated ACP clones have been shown to both significantly reduce hypertrophic gene expression and show no collagen X staining under the same conditions [22]. Furthermore, these cells have produced stable articular cartilage upon implantation in a large animal model [55]. Sub-populations of chondrocytes also need to be investigated in a focal early OA animal model to see whether they are a potential cell type for treating this clinical situation.

Previous MSC-hydrogel studies for the treatment of different diseased tissue models have shown that few MSCs remain with time in the treated area. The mechanism for in vivo MSC cartilage regeneration has been postulated to be due to the secretomic factors that initiate a paracrine signaling cascade and enable tissue repair via homing of resident cells into the tissue region and differentiating MSCs into mature chondrocytes [56,57]. Previous studies on chondrocytes (healthy and OA) and MSCs have shown that different proteomic profiles are stimulated under physioxia that can influence tissue regeneration [58]. There is also evidence that the anti-inflammatory factors released by MSCs help to reduce inflammation and restore matrix turnover in an in vitro OA cartilage and synovium explant model [59]. Further investigations into the MSC secretomic profile under physioxia and the specific metabolites that reduce inflammation and promote cartilage regeneration upon in vivo implantation are to be performed.

## 5. Conclusions

The present investigation has developed an animal model for focal early OA that can be used to evaluate cell-based regenerative therapies. Physioxic MSCs produced greater cell numbers with enhanced chondrogenic differentiation potential compared to hyperoxic MSCs. Application of physioxic MSCs in either post trauma or focal early OA defect models demonstrated a significant improvement in cartilage repair compared with hyperoxic MSCs in both models. Future investigations will seek to understand whether these results are replicated in large animal models, uncover predictive biomarkers for cartilage repair and the mechanisms involved in the regeneration process.

## Figures and Tables

**Table 1:** Summary of the groups and respective treatments for the animals used in the in vivo studies. See Figure 1 and Figure 2 for a schematic representation of the table.

Group	Experiment/Treatment	6 Weeks	12 Weeks
Healthy	No defect (right and left knee)	3 animals	
Focal early OA model	Drilled defect, medial condyle (right and left knee) and empty	3 animals	3 animal
1	Drilled defect, right medial condyle—empty		6 animals
2	Drilled defect, contralateral to group 1—cell-free hydrogel		
3	Drilled defect, right medial condyle—hyperoxic MSCs		6 animals
4	Drilled defect, contralateral to group 3—physioxic MSCs		
5	Drilled defect, time for focal early OA, right medial condyle—cleaned and empty		6 animals
6	Drilled defect, contralateral to group 5, time for focal early OA—cleaned and cell-free hydrogel		
7	Drilled defect, time for focal early OA, right medial condyle—cleaned and hyperoxic MSCs		8 animals
8	Drilled defect, contralateral to group 5, time for focal early OA—cleaned and physioxic MSCs		

## Supplementary Materials

The following are available online at https://www.mdpi.com/2079-7737/9/8/230/s1, Table S1: Summary of the OARSI scoring table for rabbit articular cartilage used to evaluate defects (adapted from Pritzker et al., 2006 and Laverty et al., 2010) [30,31]. Table S2: Summary of the Sellers score used to evaluate cartilage regeneration in treated defects (according to Sellers et al., 1997) [31].
