# Supplementary material for: Physioxia Expanded Bone Marrow Derived Mesenchymal Stem Cells Have Improved Cartilage Repair in an Early Osteoarthritic Focal Defect Model
Source: Biology (Basel). 2020 Aug 17;9(8):230. doi: 10.3390/biology9080230 (PMC7463623; doi:10.3390/biology9080230)
Supplement: Supplementary file 1 [file biology-09-00230-s001.pdf]

Table S1. Summary of the OARSI scoring table for rabbit articular cartilage used to evaluate defects (adapted from Pritzker et al, 2006 and Laverty et al, 2010) [30,31].

Grade	Key tissue features observed
0: surface and cartilage morphology intacted	Matrix: normal architecture<br>Cells: intact and appropriate orientation
1: surface intacted	Matrix: superficial zone intact, oedema and/or superficial fibrillation, focal superficial matrix condensation.<br>Cells: death, proliferation (clusters), hypertrophy, superficial zone – deeper than only the superficial zone
2: surface discontinuity	Matrix discontinuity at superficial zone (deep fibrillation); Cationic stain matrix depletion (Safranin O or Toluidine Blue) upper third of cartilage; Focal perichondronal increased stain (mid zone); Disorientation of chondron columns<br>Cells: death, proliferation (clusters), hypertrophy
3: vertical fissures (clefts)	Matrix vertical fissures into mid zone, branched fissures: Cationic stain depletion (Safranin O or Toluidine Blue) into lower two-thirds of cartilage (deep zone); New collagen formation (polarized light microscopy, Picro Sirius Red stain)<br>Cells: death, regeneration (clusters), hypertrophy, cartilage domains adjacent to fissures
4: erosion	Cartilage matrix loss: delamination of superficial layer, mid layer cyst formation<br>Excavation: matrix loss superficial layer and mid zone
5: denudation	Surface: sclerotic bone or reparative tissue including fibrocartilage within denuded surface. Microfracture with repair limited to bone surface
6: deformation	Bone remodeling (more than osteophyte formation only). Includes: microfracture with fibrocartilaginous and osseous repair extending above the previous surface

Table S2. Summary of the Sellers score used to evaluate cartilage regeneration in treated defects. (according to Sellers et al, 1997) [36].

Parameters	Key features and score	
Filling of defect relative to surface of normal adjacent cartilage	111%–125%	1
	91%–110%	0
	76%–90%	1
	51%–75%	2
	26%–50%	3
	< 25%	4
Integration of repair tissue with surrounding articular cartilage	Normal continuity and integration	0
	Decreased cellularity	1
	Gap or lack of continuity on one side	2
	Gap or lack of continuity on two sides	3
Matrix staining with Safranin O-fast green	Normal	0
	Slightly reduced	1
	Moderately reduced	2
	Substantially reduced	3
	None	4
Cellular morphology (choose first between a-b-c-d)	(a) Normal	0
	(b) Mostly round cells with the morphology of chondrocytes	
	> 75% of tissue with columns in radial zone	0
	25%–75% of tissue with columns in radial zone	1
	< 25% of tissue with columns in radial zone (disorganized)	2
	(c) 50% round cells with the morphology of chondrocytes	
	> 75% of tissue with columns in radial zone	2
	25%–75% of tissue with columns in radial zone	3
	< 25% of tissue with columns in radial zone (disorganized)	4
	(d) Mostly spindle-shape (fibroblast-like) cells	5
Architecture within entire defect (excluding margins)	Normal	0
	1–3 small voids	1
	1– 3 large voids	2
	> 3 large voids	3
	Clefts or fibrillation	4
Architecture of surface	Normal	0
	Slight fibrillation or irregularity	1
	Moderate fibrillation or irregularity	2
	Severe fibrillation or disruption	3
Percentage of new subchondral bone	90%–100%	0
	75%–89%	1
	50%–74%	2
	25%–49%	3
	< 25%	4
Formation of tidemark	Completed	0
	75%–99%	1
	50%–74%	2
	25%–49%	3
	< 25%	4
